# Ultra-Processed Foods and Human Health: A Systematic Review and Meta-Analysis of Prospective Cohort Studies

**DOI:** 10.1016/j.advnut.2023.09.009

**Published:** 2023-12-18

**Authors:** Marilena Vitale, Giuseppina Costabile, Roberta Testa, Giovanna D’Abbronzo, Immacolata Cristina Nettore, Paolo Emidio Macchia, Rosalba Giacco

**Affiliations:** 1 Department of Clinical Medicine and Surgery, Diabetes, Nutrition and Metabolism Unit, Federico II University of Naples, Italy; 2 Department of Clinical Medicine and Surgery, Federico II University of Naples, Italy; 3 Institute of Food Sciences, National Research Council, Avellino, Italy

**Keywords:** ultra-processed foods, diabetes, hypertension, dyslipidemia, obesity, human

## Abstract

Evidence of associations between ultra-processed foods (UPF) and increased risk of cardiovascular disease is emerging, but it is unclear how much this is influenced by the methodology used to assess the UPF intake or by the level of consumption. We conducted a meta-analysis to evaluate 1) the association between UPF consumption and risk of diabetes, hypertension, dyslipidemia, and obesity, using prospective cohort studies; 2) the differential associations depending on the methodology used to assess UPF intake and the level of UPF consumption and 3) the quality of evidence using the NutriGrade scoring system. A systematic literature search was conducted in PubMed/MEDLINE, ISI Web of Science, and Scopus through 1 April, 2023, on studies conducted in humans providing data for the highest compared with the lowest UPF consumption categories. Summary relative ratios (RRs) and 95% confidence intervals (95% CI) were estimated using a random-effects model. Out of 4522 articles retrieved from the literature search, 25 reports met the criteria for inclusion in the meta-analysis, 7 for diabetes, 5 for hypertension, 3 for dyslipidemia, and 13 for obesity. A consistently positive association between high UPF intake and increased risk of developing diabetes (37%), hypertension (32%), hypertriglyceridemia (47%), low HDL cholesterol concentration (43%), and obesity (32%) was observed, even if the quality of evidence was not satisfying. However, these risks varied significantly depending on the methodology used to assess UPF consumption, with a difference of more than 50% between the methods. Based on the level of intake, we did not observe significant differences in the results. These findings show that UPF consumption is associated with higher risk of diabetes, hypertension, dyslipidemia, and obesity, but the level of risk consistently changes depending on the methodology used to assess UPF intake. Therefore, caution should be used when interpreting and extrapolating the results.

**Statement of Significance**

Consumption of ultra-processed foods (UPF) has been shown to negatively impact human health with long-term effects on cardiometabolic health, increasing risk of obesity, type 2 diabetes, dyslipidemia, and cardiovascular disease (CVD).

These data have several limitations due to the meta-evidence of low quality and the fact that the level of risk consistently changes depending on the methodology used to assess UPF intake. Therefore, caution should be used when interpreting and extrapolating the results.

## Introduction

Ultra-processed foods (UPF) are ready-to-eat or ready-to-heat industrial preparations made largely or entirely with substances extracted from food, often chemically modified, with additives, and with a small proportion of whole food [1]. Therefore, the term UPF includes soft drinks, packaged snacks, sugared breakfast cereals, cookies, processed meats, and packaged frozen or shelf-stable meals, but also flavored yogurts, low-calorie or low-fat products, breakfast cereals, and products "fortified" with beneficial nutrients [1].

To date, consumption of UPF has been shown to negatively impact human health by adversely affecting body weight, insulin resistance, systemic inflammation, blood pressure, and gut microbiota [[2], [3], [4]]. Recent epidemiological studies also suggest that higher consumption of UPF has long-term effects on cardiometabolic health, increasing risk of obesity, type 2 diabetes, dyslipidemia, and cardiovascular disease (CVD) [5]. A positive association was consistently found in these studies, although risk values varied widely by cohort and level of total UPF intake.

In the Framingham Offspring Study, after 18 y of follow-up, each additional daily serving of UPF was associated with a 7% increased risk of CVD [6]. Similarly, in the French NutriNet-Santé cohort study, increased consumption of UPF was associated with a 12% increased risk of CVD [7]. Prospective cohort data on the association between total UPF consumption and type 2 diabetes also show a positive and strong association with a 15% to 53% higher risk of developing type 2 diabetes, depending on the study cohort and UPF consumption [[8], [9], [10], [11]].

It is noteworthy that the available meta-analysis on the effects of UPF on cardiometabolic health in humans considers cohorts in which there is considerable overlap between the categories of low and high UPF intake. More in detail, the same amount of UPF is considered high in some cohorts and low in others. From a biological and mechanistic perspective, an accurate comparison of results is not possible because countries in which the energy component of UPF represents up to 80% of the diet have a completely different dietary profile than countries in which intake is much lower because of consumption of more nutritionally adequate foods.

In addition, the methods used to assess eating habits and the approach used to categorize foods into the 4 groups of NOVA classification also vary widely across cohorts. Eating habits are often assessed using 24-h recall or food intake frequency questionnaires. The 24-h recall's limitations include the inability to account for diurnal variation and, therefore, to estimate the usual distribution of food intake, so it may not be representative of habitual diet. The FFQ's limitations include an inability to quantify the amount of food consumed, making them less accurate, recall bias due to the length of recall period, and errors due to the different number of food lists between the studies. These methodological differences may impact the magnitude of disease risk associated with UPF consumption.

Therefore, in this systematic review and meta-analysis, we first assessed the association of total UPF consumption on the occurrence of major cardiovascular disease risk factors, i.e., diabetes, hypertension, dyslipidemia, and obesity, using prospective cohort studies. We then performed a sensitivity analysis to account for possible differential associations depending on the methodology used to assess UPF intake and the level of total UPF consumption. Finally, we assessed the quality of this meta-evidence using the NutriGrade scoring system [12].

## Methods

### Search strategy

This study was conducted according to the 2020 Preferred Reporting Items for Systematic Reviews and Meta-Analyses (PRISMA) guidelines [13]. A systematic literature search was conducted in PubMed/MEDLINE, ISI Web of Science, and Scopus through April 1, 2023, with no language or date restrictions. Search terms were a combination of free-text terms and controlled vocabulary related to UPF and cardiovascular disease risk factors, including (ultra-processed food OR ultraprocessed food OR ultra processed OR processed food OR NOVA OR nova food classification OR nova food OR NOVA food classification system) AND (intake OR consumption OR eating) AND (obesity OR overweight OR body weight OR blood pressure OR hypertension OR systolic OR diastolic OR inflammation OR dyslipidemia OR LDL-cholesterol OR HDL cholesterol OR triglycerides OR cholesterol OR diabetes mellitus OR type 2 diabetes mellitus OR diabetes). The reference lists of retrieved articles were manually searched for additional studies. Both longitudinal and cross-sectional studies were included. The present study protocol was submitted to the International Prospective Register of Systematic Reviews database (PROSPERO) under registration number CRD42023418668.

### Inclusion and exclusion criteria

Studies that met the following criteria were included in this analysis: 1) observational (cohort, case-control, or cross-sectional); 2) adults aged ≥18 y; 3) published in English; 4) data on the association between UPF consumption and cardiovascular disease risk factors (i.e., diabetes, hypertension, dyslipidemia, and obesity); 5) effect estimates in terms of hazard ratio (HR), relative risk (RR), or odds ratio (OR) with 95% confidence interval (95% CI); and 6) outcomes adjusted for covariates. Studies conducted in children and adolescents (<18 y), reviews, conference letters, notes, reports, short surveys, unpublished studies, and case reports were excluded. The population, intervention, comparison group, and outcome are shown in Supplemental Table 1.

### Study selection

Studies were selected by screening titles and abstracts, followed by individual assessment of all potentially relevant full-length articles by 2 reviewers (M.V. and G.C.). Disagreements regarding the inclusion and exclusion of selected articles were resolved by consensus or discussion or by the involvement of a third researcher (R.G.). Studies were excluded if they did not meet the above criteria. If >1 study was published on the same cohort, only the results of the most recent study were included in the analysis. For studies with missing data, authors were contacted for data collection when possible.

### Data extraction and study quality

Two investigators (M.V. and G.C.) independently reviewed each eligible study. The following information was extracted: 1) name of first author, 2) year of publication, 3) country, 4) definition of UPF, 5) method of assessing dietary intake, 6) main foods contributing to UPF consumption, 7) mean/median or range of intake in each category, 8) number of participants, 9) number of cases, 10) mean/median duration of follow-up, 11) covariates used for adjustments in the multivariable analyses, 12) outcome measures, 13) age, 14) gender, and 15) BMI. If the number of participants/person-y and cases was not provided, the corresponding authors were contacted for further information.

Two investigators (M.V. and G.C.) assessed the quality of each study separately using the Newcastle-Ottawa Scale (NOS) [14], which assessed the representativeness of the exposed cohort, the selection of the unexposed cohort, the identification of exposure, the evidence that the outcome of interest was not available at baseline and comparability of the cohort, assessment of the outcome, whether the follow-up period was long enough to capture the outcome, and assessed the adequacy of the follow-up of the cohort (details are provided in Supplemental Table 2). Scores ranged from 0 to 9, with a higher score indicating higher study quality.

### Data synthesis and analysis

In this meta-analysis, UPF were considered the main exposure of interest. UPF were defined according to the NOVA classification system [1]. More specifically, UPF were defined as industrially produced, ready-to-eat, or ready-to-heat preparations that contain few whole foods [1]. These foods include soft drinks, packaged snacks, sugared breakfast cereals, cookies, processed meats, and packaged frozen or shelf-stable meals, but also flavored yogurts, low-calorie or low-fat products, breakfast cereals, and products "fortified" with beneficial nutrients.

Relative risks were used as a common measure of association between studies. Studies conducted in 2 independent cohorts were treated as separate reports. We used the forest plots to evaluate RRs and 95% CIs of outcomes in the groups with high and low UPF consumption, respectively. Because of the large heterogeneity in the fixed-effects models, random-effects meta-analyses based on the I2 cut-off were performed for all comparisons. The inverse variance method was used to determine the weights of the studies. Heterogeneity between studies was estimated using the Cochran Q test (*P* < 0.05, indicating statistically significant heterogeneity) and the I^2^ statistic. It was suggested that I^2^ statistics of 0 to 25%, 25 to 50%, 50 to 75%, and 75 to 100% indicate modest, modest-to-moderate, moderate-to-strong, and strong heterogeneity, respectively [14]. Additional sensitivity analyses were planned and performed in advance by systematically omitting 1 study at a time and recalculating the summary association to test the robustness of the results and the impact of individual studies on heterogeneity. In addition, specific sensitivity analyses were also performed to account for the possible differential association based on the methodology used to assess UPF intake (FFQ vs. 24-h food recall) and the level of total UPF consumption.

Funnel plots were presented to visually identify publication bias. Publication bias was also tested using Egger's regression symmetry test, with significant bias detected at *P* < 0.10 [15].

All statistical analyses were performed using RevMan 5.4 (Review Manager RevMan-Computer program; version 5.4; The Cochrane Collaboration, 2020) and R 4.1.0 (The R Project for Statistical Computing; version 4.1.0, 2021), and all tests were 2-sided with a significance level of 0.05 unless otherwise stated.

The NutriGrade scoring system was used to assess the quality of our meta-analyses. This scoring system was derived from the Grading of Recommendations Assessment, Development, and Evaluation and is more appropriate for nutrition research. The scoring system assessed risk of bias, precision, heterogeneity, directness, publication bias, funding bias, effect size, and dose-response for meta-analyses of cohort studies (details are provided in Supplemental Table 3). The meta-analysis was classified as of high, moderate, low, or very low quality if it scored 8 to 10, 6 to < 8, 4 to < 6, and 0 to < 4 points, respectively [12].

## Results

**FIGURE 1:**
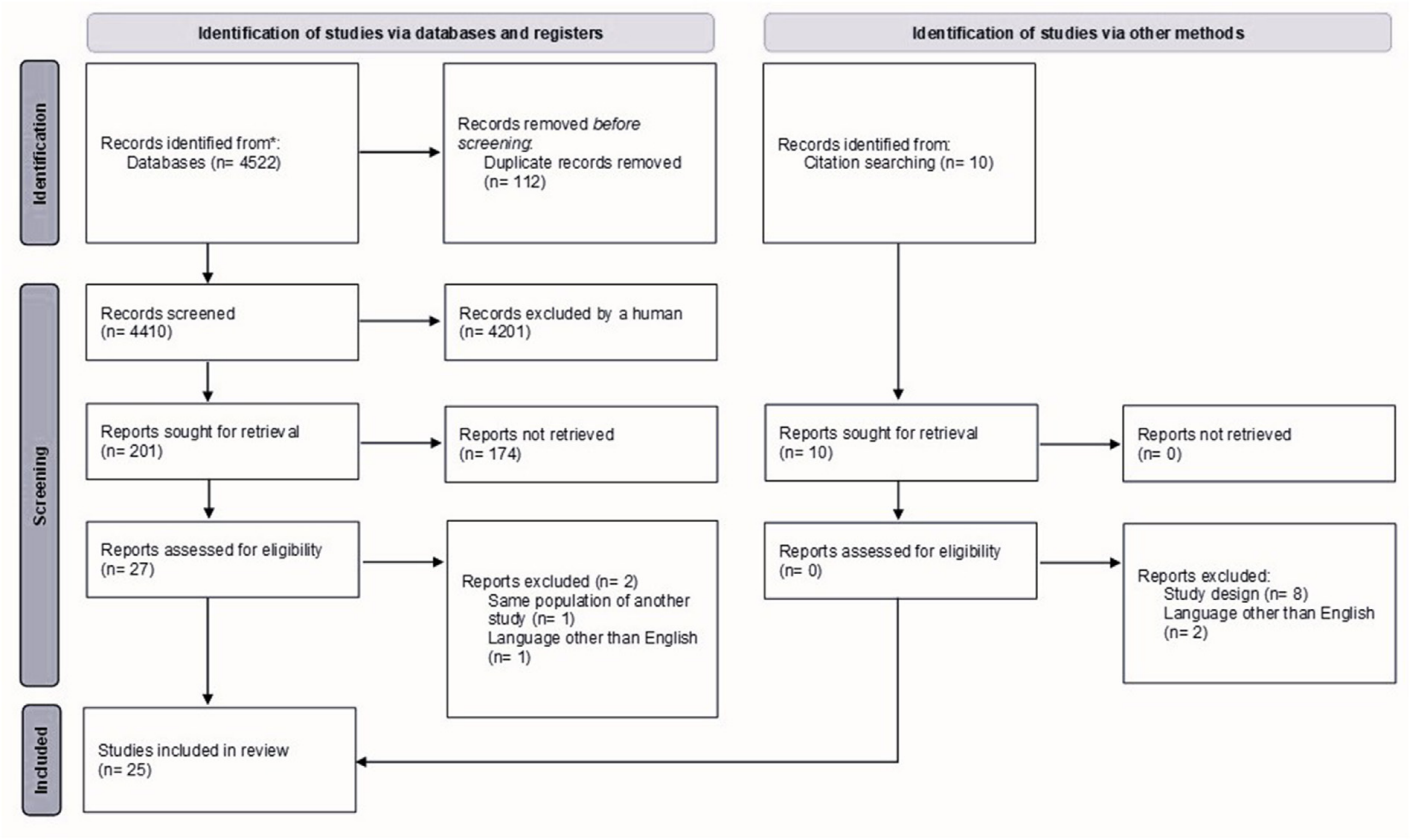
PRISMA flowchart indicating the results of the search strategy. From: M. J. Page, J. E. McKenzie, P. M. Bossuyt, I. Boutron, T. C. Hoffmann, C. D. Mulrow, et al. The PRISMA 2020 statement: an updated guideline for reporting systematic reviews, BMJ 2021;372:n71. https://doi.org/10.1136/bmj.n71. For more information, visit: http://www.prisma-statement.org/

We found 4522 studies through a database search and reference lists. After removing duplicates, 4410 records remained (Figure 1). The titles and abstracts of these studies were reviewed, but 4242 studies were excluded because they did not classify UPF based on NOVA classification and 143 because they did not meet our inclusion criteria. Subsequently, 27 full-text studies were assessed; one article was excluded because it included the same population study as another included study, and one article was not in English. Finally, 25 studies met our inclusion criteria and were considered for quantitative analysis in this paper.

### Study characteristics

The main characteristics of the identified studies are shown in Supplemental Table 1. The included studies comprised 2959 cases of diabetes with a follow-up of 248,534 person-y, 2177 cases of hypertension with a follow-up of 33,508 person-y, 4934 cases of dyslipidemia with a follow-up of 7146 person-y, and 124,155 cases of obesity with a follow-up of 525,122 person-y. Follow-up time ranged from 2.0 to 14 y. Of the 25 included studies, 9 were conducted in the United States [[16], [17], [18], [19], [20], [21], [22], [23], [24]], 10 in Europe [2,5,[7], [8], [9], [10],[25], [26], [27], [28]], and 6 in Asia [[29], [30], [31], [32], [33], [34]]. The UPF intake was assessed using 3 different methods: validated long-form FFQs in 12 studies [5,8,9,16,[18], [19], [20],^22^,^23^,^25^,^29^,^34^], 24-h dietary recall in 12 studies [2,10,11,17,21,24,26,27,[30], [31], [32], [33]], and only 1 study (28) used a 4-d food diary. The amount of UPF was reported as grams per d in 4 studies [8,16,25,30], as a percentage of daily energy intake in 15 studies [2,5,[17], [18], [19], [20], [21], [22], [23], [24],^28^,^29^,^31^,^33^,^34^], and as a proportion relative to total dietary weight in 6 studies [9,10,18,26,27,32]. In the low exposure group, amounts of UPF ranged from 0 or nonconsumers to 214.6 g/d or 38.5% Kcal/d; in the high exposure group, amounts ranged from 50 g/d or 20.4% Kcal/d to 686 g/d or 74.2% Kcal/d with very high standard deviations. The main foods contributing to UPF intake were not reported in all included studies; however, studies that described in more detail the dietary quality of individuals consuming more UPF showed that soft drinks, bakery products, processed meats, and meat products contributed most to the total consumption of UPF.

Most included studies were classified as high quality using the Newcastle-Ottawa Quality Assessment Scale (>8), and the mean quality scores of the studies were 8.1, 8.0, 7.8, 8.3, and 7.8 for diabetes, dyslipidemia, hypertension, low HDL cholesterol, and obesity, respectively (Supplemental Table 2).

### UPF consumption and diabetes incidence

**FIGURE 2:**
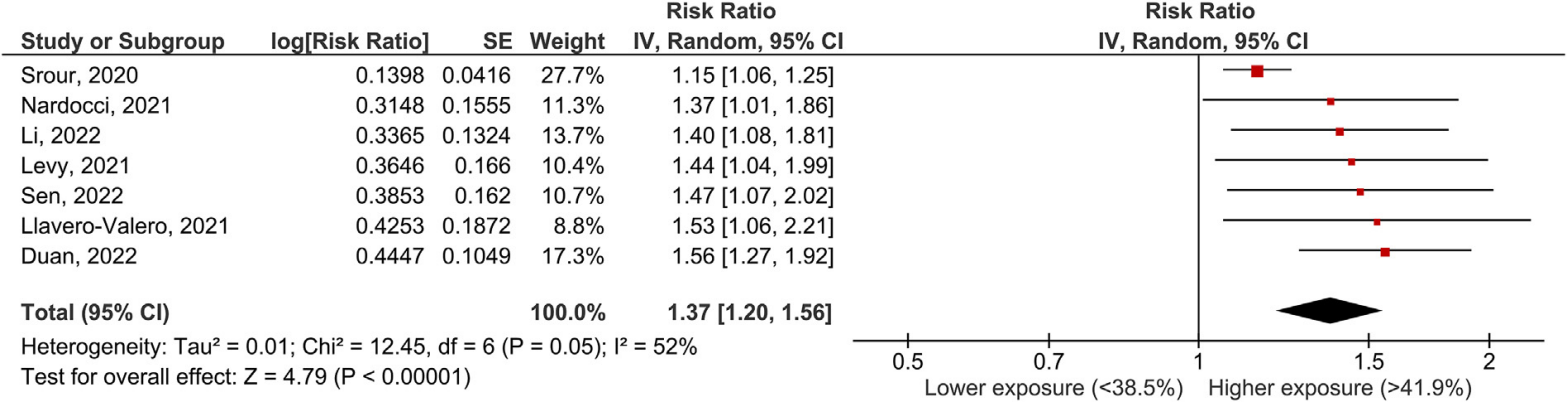
UPF consumption and diabetes incidence

Seven articles [[8], [9], [10], [11],^16^,^17^,^32^] with 7 cohorts were included in the analysis of UPF consumption and diabetes incidence. The mean follow-up time for the pooled studies was 8.0 y (range 3.5 to 14 y). The pooled RR of diabetes incidence for the highest versus lowest category of UPF was 1.37 (95% CI: 1.20, 1.56), with moderate heterogeneity (I^2^ = 52%, *P* = 0.05) (Figure 2). By systematically omitting 1 study at a time, heterogeneity was generated by 2 studies [9,11], and when these reports were excluded, the association remained statistically significant without significant heterogeneity.

Sensitivity analyses were performed to account for differences in RR based on the methodology used to assess UPF consumption (validated long-form FFQ vs. 24-h dietary recall) and the amount consumed. Of the 7 included articles, 3 used a validated FFQ [8,9,16], and 4 used the 24-h dietary recall [10,11,17,32]. The amount of UPF was reported as grams per d in 2 studies [8,16], as a proportion of total dietary weight in 2 studies [9,10], and as a percentage of daily energy intake in 1 study [17]. Two studies reported a hugely different amount of UPF compared with the other studies and were therefore excluded from the sensitivity analyses [11,32]. The pooled RR of diabetes incidence for the highest versus lowest category of UPF was 1.53 (95% CI: 1.31, 1.79) with no significant heterogeneity (I^2^ = 0%, *P* = 0.95) and 1.25 (95% CI: 1.11, 1.42) with moderate but no significant heterogeneity (I^2^ = 30%, *P* = 0.23) when we considered the methodology used to assess UPF consumption (FFQ or 24-h dietary recall). On the basis of the amount consumed, the pooled RR of diabetes incidence for the highest versus lowest category of UPF was 1.50 (95% CI: 1.18, 1.90) with no significant heterogeneity (I^2^ = 0%, *P* = 0.87) and 1.52 (95% CI: 1.28, 1.81) with no significant heterogeneity (I^2^ = 0%, *P* = 0.68) (grams per d or proportion relative to total dietary weight).

According to the NutriGrade scoring system, the quality of evidence for the association between consumption of UPF and the incidence of diabetes was moderate (Table 1).

**TABLE 1:** NutriGrade scoring system

Outcome	Quality of evidence	Mean
Diabetes	Moderate	6.21
Hypertension	Low	5.50
Hypertriglyceridemia	Low	5.67
Low-HDL-c		
Obesity	Low	6.00

### UPF consumption and incidence of hypertension

**FIGURE 3:**
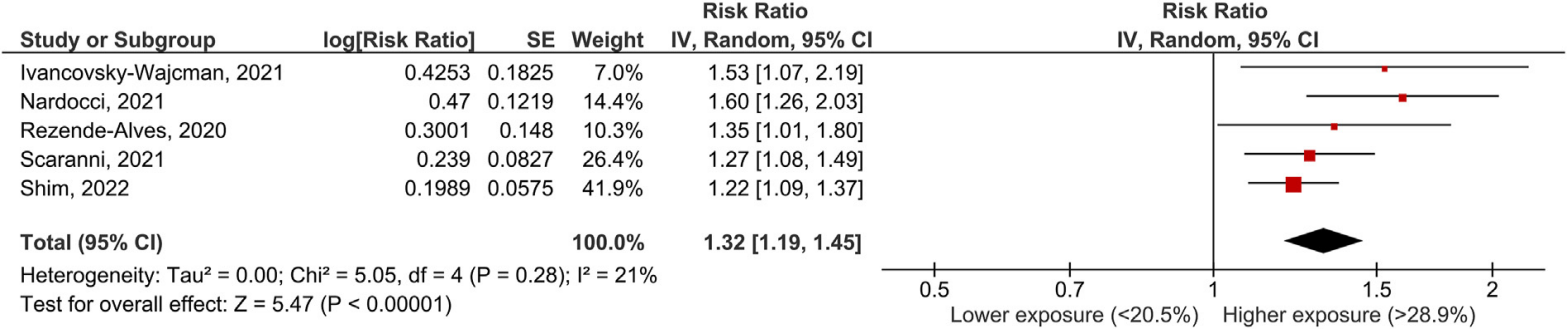
UPF consumption and incidence of hypertension

Five articles [17,19,20,33,34] with 5 cohorts were included in the analysis of UPF consumption and the occurrence of hypertension. The mean follow-up time for the pooled studies was 3.0 y (range, 2.0 to 3.9 y). The pooled RR of the incidence of hypertension for the highest versus lowest category of UPF use was 1.32 (95% CI: 1.19, 1.45), with no significant heterogeneity (I^2^ = 21%, *P* = 0.28) (Figure 3).

Sensitivity analyses were performed to account for differences in RR based on the methodology used to assess UPF consumption (validated long-form FFQ vs. 24-h dietary recall) and the amount consumed. Of the 5 included articles, 3 used a validated FFQ [19,20,34], and 2 used the 24-h dietary recall (17,33). The amount of UPF was reported as a percentage of daily energy intake in all studies. The pooled RR of the incidence of hypertension for the highest versus the lowest category of UPF was 1.32 (95% CI: 1.16, 1.50) with no significant heterogeneity (I^2^ =0 %, *P* = 0.64) and 1.37 (95% CI: 1.05, 1.78) with strong and significant heterogeneity (I^2^ = 75%, *P* = 0.04) when the methodology to assess UPF consumption (FFQ or 24-h dietary recall) was taken into account. On the basis of consumption level, the pooled RR of the incidence of hypertension for the highest versus the lowest category of UPF was 1.49 (95% CI: 1.24, 1.80) with no significant heterogeneity (I^2^ = 0%, *P* = 0.38) and 1.31 (95% CI: 1.13, 1.52) with no significant heterogeneity (I^2^ = 0%, *P* = 0.35) (high vs. low consumption levels, respectively).

According to the NutriGrade scoring system, the quality of evidence for the association between consumption of UPF and the occurrence of hypertension was low (Table 1).

### UPF consumption and incidence of dyslipidemia

**FIGURE 4:**
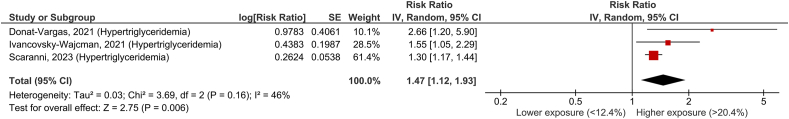
UPF consumption and incidence of hypertriglyceridemia

Three articles [5,18,34] with 3 cohorts were included in the analysis of consumption of UPF and incidence of hypertriglyceridemia. The mean follow-up time for the pooled studies was 5.5 y (range, 3.9 to 7.0 y). The pooled RR for the incidence of hypertriglyceridemia for the highest versus lowest category of UPF consumption was 1.47 (95% CI: 1.12, 1.93), with no significant heterogeneity (I^2^ = 46%, *P* = 0.16) (Figure 4). Because of the small number of studies included in this meta-analysis, it was not possible to perform a sensitivity analysis to account for possible differential associations depending on the methodology used to assess UPF intake and the level of total consumption of UPF extension.

According to the NutriGrade scoring system, the quality of evidence supporting the association between UPF consumption and the occurrence of hypertriglyceridemia was low (Table 1).

**FIGURE 5:**
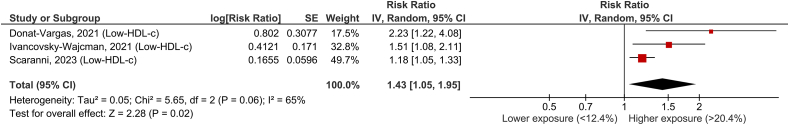
UPF consumption and occurrence of low HDL cholesterol

Three articles (5,18,34) with 3 cohorts were included in the analysis of UPF consumption and the occurrence of low HDL cholesterol. The mean follow-up time for the pooled studies was 5.5 y (range, 3.9 to 7.0 y). The pooled RR for the occurrence of low HDL cholesterol for the highest versus lowest category of UPF consumption was 1.43 (95% CI: 1.05, 1.95), with heterogeneity that was statistically significant (I^2^ = 65%, *P* = 0.06) (Figure 5). Because of the small number of studies included in this meta-analysis, it was not possible to perform a sensitivity analysis to account for possible differential associations based on the methodology used to assess UPF intake and the level of total consumption of UPF extension.

According to the NutriGrade scoring system, the quality of evidence for the association between UPF consumption and the occurrence of low HDL cholesterol was low (Table 1).

The association with total cholesterol or LDL-cholesterol was not considered because of a lack of data.

### UPF consumption and obesity incidence

**FIGURE 6:**
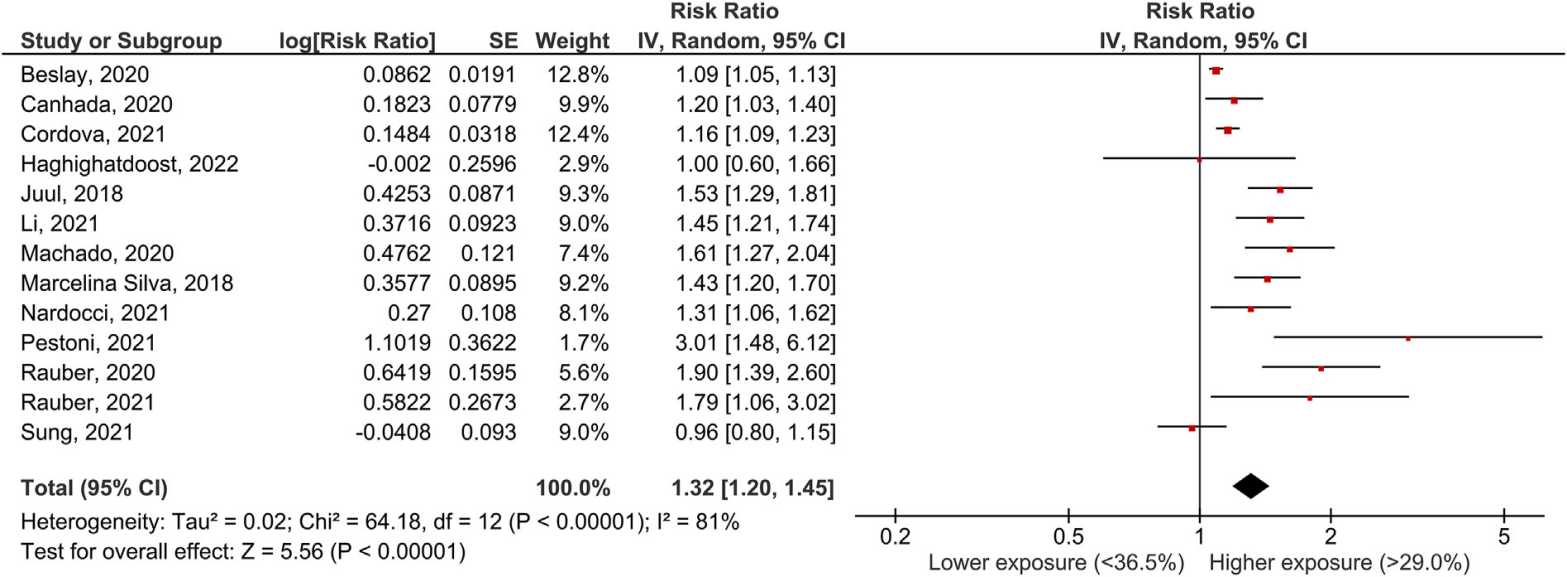
UPF consumption and obesity incidence

Thirteen articles [2,17,[21], [22], [23], [24], [25], [26], [27], [28], [29], [30], [31]] with 13 cohorts were included in the analysis of UPF consumption and obesity incidence. The mean follow-up time for the pooled studies was 6.9 y (range 5.0 to 12.0 y). The pooled RR of the incidence of obesity for the highest versus lowest category of UPF use was 1.32 (95% CI: 1.20, 1.45), with large heterogeneity (I^2^ = 81%, *P* < 0.0001) (Figure 6). By systematically omitting 1 study at a time, heterogeneity remained statistically significant.

Sensitivity analyses were performed to account for differences in RR based on the methodology used to assess UPF consumption (validated long-form FFQ vs. 24-h dietary recall) and the amount consumed. Of the 13 included articles, 4 used a validated FFQ [22,23,25,29], 8 used the 24-h dietary recall [17,21,24,[26], [27], [28],^30^,^31^], and only 1 study (2) used a 4-d food diary. The amount of UPF was reported as grams per d in 3 studies [25,27,30] and as a percentage of daily energy intake in 10 studies [2,17,[21], [22], [23], [24],^26^,^28^,^29^,^31^]. The pooled RR of the incidence of obesity for the highest versus lowest category of UPF was 1.22 (95% CI: 1.10, 1.35) with moderate but nonsignificant heterogeneity (I^2^ = 44%, *P* = 0.15) and 1.40 (95% CI: 1.18, 1.66) with strong and significant heterogeneity (I^2^ = 87%, *P* < 0.0001) when we used the methodology used to assess UPF consumption (FFQ vs. 24-h dietary recall). Based on the amount consumed, the pooled RR of obesity incidence for the highest versus lowest category of UPF was 1.60 (95% CI: 1.38, 1.84) with moderate but nonsignificant heterogeneity (I^2^ = 36%, *P* = 0.17) (proportion relative to total dietary weight).

According to the NutriGrade scoring system, the quality of evidence for the association between consumption of UPF and the occurrence of obesity was low (Table 1).

### Publication bias and quality of evidence

Publication bias was assessed using funnel plots (see Supplemental Figure 1). Visual analysis of the funnel plots indicated that the associations between UPF and risk of metabolic disorders (i.e., diabetes, hypertension, hypertriglyceridemia, low HDL cholesterol, obesity) were symmetric and, thus, at minimal risk for publication bias. This was confirmed by Egger's linear regression test.

According to the NutriGrade scoring system, the quality of evidence for the association between consumption of UPF and the incidence of diabetes was moderate, and the quality of evidence for the association between consumption of UPF and hypertension, hypertriglyceridemia, low HDL cholesterol concentration, and obesity was low (Table 1).

## Discussion

According to the available literature on UPF, our study shows that overall consumption of UPF is associated with an increased incidence of major cardiovascular disease risk factors, i.e., diabetes, hypertension, dyslipidemia, and obesity, based on data from prospective cohort studies.

Specifically, the results of our meta-analyses consistently show a positive association between high UPF intake and an increased risk of developing diabetes (37%), hypertension (32%), hypertriglyceridemia (47%), low HDL cholesterol (43%), and obesity (32%). Of note, these associations were derived from data adjusted for several potential confounders (i.e., general population characteristics, dietary habits, country, etc.).

Thus, in agreement with previous meta-analyses [[35], [36], [37], [38], [39]], our results further confirmed the strong positive association between UPF intake and health risk. With regard to the incidence of diabetes, Chen et al. [39] found in a meta-analysis based on 5 prospective cohort studies that high UPF consumption, expressed as a percentage of grams UPF/d, was associated with a 40% higher risk of developing diabetes. In addition, in the same study, authors performed another analysis where the total UPF intake was expressed for each 10% increment of consumption; in this case, risk of developing type 2 diabetes was equal to 12%. Another meta-analysis by Moradi et al. [38] based on 5 studies found that participants with high UPF intake had a 74% higher risk of diabetes. Wang et al. [35], focusing on hypertension, found that higher consumption of UPF increased risk of hypertension in adults by 23%. In another meta-analysis by Pagliai et al. [37], no statistically significant association was found between the highest consumption of UPF and hypertension. Regarding obesity, our findings of a positive association between higher intake of UPF and obesity are consistent with previous studies. A recent meta-analysis reported that participants with higher intakes of UPF had a greater risk of overweight and obesity than participants with lower intakes of UPF [36].

However, given the limited number of studies included in the meta-analyses, the large heterogeneity, the fact that all studies were conducted in industrialized countries, the considerable overlap between the categories of low and high UPF intake, and the low quality of meta-evidence assessed using the NutriGrade scoring system, caution should be exercised when interpreting and extrapolating the results. This is particularly true for the association between UPF consumption and the occurrence of hypertriglyceridemia and low HDL cholesterol, as the number of included studies was small (only 3), but also for the occurrence of hypertension and obesity, as the quality of included studies was low. In contrast, the association between UPF consumption and the incidence of type 2 diabetes is quite robust, as it is based on studies of moderate number and quality, and the significant heterogeneity disappeared when 2 of the 7 studies were excluded from the analysis.

Compared with the methodology of previous studies, we not only performed a categorical meta-analysis but also assessed the different associations between UPF intake and outcomes, considering the methodology used to assess UPF consumption and the level of intake. In this regard, the sensitivity analysis we performed confirmed all positive associations while reducing heterogeneity between studies. However, risk of developing diabetes, hypertension, hypertriglyceridemia, low HDL cholesterol concentration, and obesity varied significantly depending on the methodology used to assess UPF consumption. Based on a pooled RR for diabetes of 1.37, sensitivity analysis yielded a risk of 1.53 when FFQ was used and 1.25 when UPF intake was based on 24-h recall, with a difference of more than 50% between the 2 methods used to assess UPF intake. A similarly significant difference between the 2 methods was found with respect to risk of developing obesity. Based on a pooled RR for obesity of 1.32, sensitivity analysis showed a risk of 1.22 when the FFQ was used and 1.40 when UPF intake was based on the 24-h recall. For hypertension, the difference between the results obtained with the 2 assessment methods was < 5%, mainly because of the small number of studies included in the analysis.

Regarding the assessment of the level of intake of UPF, we did not observe significant differences in the results when we performed a sensitivity analysis considering whether the intake was expressed in g/d or corrected for energy intake. In fact, the difference between the 2 methods was greater than 10% only in the case of hypertension.

Therefore, the results of the sensitivity analyses emphasize the importance of the method used to assess UPF consumption but not that used to assess the level of intake.

Recently, several potential mechanisms have been proposed to explain the association between UPF intake and risk of diabetes, hypertension, dyslipidemia, and obesity. As mentioned earlier, UPF are nutritionally imbalanced due to their high content of free/added sugars, saturated and trans fatty acids, and low content of protein, fiber, and micronutrients. These differences in the nutrient content of UPF may play a key role in explaining their negative effects on health.

In more detail, the high content of refined carbohydrates and free sugar, components rapidly absorbed into the bloodstream, increases the glycemic load, the postprandial glucose and insulin responses, and worsens the insulin resistance, all factors that negatively influence the cardiovascular profile. In addition, the high saturated fat content contributes to worse postprandial lipemia and increases total cholesterol and LDL-cholesterol concentrations. Also, the low fiber content contributes to the negative effects of UPF consumption due to suppression of the sensation of hunger and an increase of the feeling of satiety, leading to a long-term increase in daily energy intake and consequent weight gain. In addition, several additives commonly found in UPF (i.e., heterocyclic amines, acrylamide, polychromatic hydrocarbons, and furans) can impair the functionality of the endocrine system and the microbiome, increasing the incidence of adverse health outcomes [40]. In particular, these additives promote inflammatory diseases with potentially important effects on body weight, adiposity, and the development of type 2 diabetes [[41], [42], [43]]. Finally, people who eat unhealthy diets are likely to have unhealthy lifestyles [44] (including excessive smoking and drinking, and lack of physical activity), which are also high-risk factors for adverse cardiovascular events and deaths [[45], [46], [47]].

This meta-analysis has several limitations. First, it does not allow for clear and robust conclusions about the role of UPF on human health because the results are strongly influenced by the following methodological limitations of the included studies: 1) differences in the level of UPF intake, expressed in some as g/d, in others as a percentage of the total weight of food ingested, and still in others as a percentage of total daily energy intake; 2) the lack of standardization of the value to define a population with high or low exposure; in fact, the value defined as high in one study is reported as low in another. Second, most of the studies included do not specify the kind of foods contributing to the UPF intake, i.e., soft drinks, packaged snacks, sugared breakfast cereals, cookies, processed meats, and packaged frozen or shelf-stable meals, flavored yogurts, low-calorie or low-fat products, breakfast cereals. This is a critical point because the categories of UPF can vary widely among participants and have different effects on risk of developing diabetes, hypertension, dyslipidemia, and obesity. In our meta-analysis, of the 7 studies including diabetes, only 5 reported on the categories of UPF, whereas only 1 study reported on obesity and no study reported on hypertension and dyslipidemia. Another methodological limitation lies in the NOVA classification because, as mentioned above, UPF also includes nutritionally healthy foods such as yogurt, cereals, and so on, which could have a positive impact on health. Finally, the lack of data to evaluate the dose-response analysis does not allow clear and robust conclusions.

To the contrary, the study also has some strengths, such as a rigorous search and selection strategy that identified all available prospective cohort studies that examined the association between UPF consumption and health status and the fact that we performed a sensitivity analysis to evaluate the different level of risk, depending on the methodology used, to assess UPF consumption.

## Conclusion

The results of our meta-analysis show that total UPF consumption is associated with higher risk of diabetes, hypertension, dyslipidemia, and obesity. However, according to the NutriGrade scoring system, the meta-evidence we obtained is of low quality. In addition, the data from the sensitivity analysis also show that the level of risk consistently changes depending on the methodology used to assess UPF intake. Therefore, caution should be used when interpreting and extrapolating the results.

Overall, our study supports current recommendations to limit total UPF consumption. However, to assess the health effects of UPF more accurately, new tools should be used to assess UPF consumption that can specify food classes, the nutrient composition of UPF foods, and the specific processes or additives used in their production.

## Author contributions

The authors’ responsibilities were as follows—M.V. and R.G. are responsible for study design; M.V., G.C., I.C.N., P.E.M., and R.G. are responsible for writing; M.V., I.C.N., P.E.M., and R.G. have primary responsibility for the final content. All authors have read and approved the final manuscript.

## Conflicts of interest

All authors have no conflict of interest to declare.

## Funding

The present analyses have been supported by a research grant from MUR (Project ALIFUN ARS01_00783). The funder had no role in the study design, collection, analysis, and interpretation of data, in the writing of the manuscript and in the decision to submit the article for publication.

## Declaration of interests

The authors declare that they have no known competing financial interests or personal relationships that could have appeared to influence the work reported in this paper.

## Appendix A Supplementary data

Multimedia component1 Multimedia component1
