# Supplementary material for: Ultra-Processed Foods and Human Health: A Systematic Review and Meta-Analysis of Prospective Cohort Studies
Source: Adv Nutr. 2023 Dec 18;15(1):100121. doi: 10.1016/j.advnut.2023.09.009 (PMC10831891; doi:10.1016/j.advnut.2023.09.009)

**Ultra-processed foods and human health: a systematic review and meta-analysis of prospective cohort studies**

Marilena Vitale

**Supplementary Table 1.** Characteristics of the prospective cohort studies included in the analysis.

| **Authors,**  **years** | **Outcome** | **Coutry/**  **Ethnicity** | **Type of foods** | **Dietary intake assessment: method** | **Main food contibutors** | **Follow-up**  **(years)** | **Total population (number of cases)** | **Higher exposure** | **Lower exposure** |
| --- | --- | --- | --- | --- | --- | --- | --- | --- | --- |
| Duan,  2022 | Diabetes | Netherlands | UPFs intake as (weight %) | 110-item FFQ | Of all UPF groups, staple/starchy food and cereals like sliced bread or granola (22.1%), non-cheese dairy products like chocolate milk and ice cream (13.7%), and sugary beverages like lemonade or ice tea (9.7%) contributed most to the overall intake of UPFs | 41 months | 70421 (1128) | 48.7 (45.2, 53.9) | 23.7 (20.3, 26.0) |
| Li,  2022 | Diabetes | China | UPFs intake as g/day (weight%) | 24-h dietary recall on each of 3 consecutive days | Not reported | 14 years | 12849 (267) | >50 g/d (10.4±6.8%) | none |
| Sen,  2022 | Diabetes | Canadian province of Quebec | UPFs intake as g/day | 164-item FFQ | Soft and isotonic drinks, fast food and ready to eat and cookies, biscuits, muffins,  and cake food groups were the main food groups contributing to the total of UPFs | 7 years | 3880 (263) | 579.5±407.0 g/d | 107.1±33.9 g/d |
| Levy,  2021 | Diabetes | UK | UPFs intake as (%) | 24-h dietary recall | The most commonly consumed UPF were beverages (8.6% of total g/day), bakery products and breakfast cereals (6.6% of total g/day) and industrial-processed frozen/self-stable prepared meals and salty snacks (4.4% of total g/day) | 5.4 years | 23009 (305) | 41,9% of total g/day | 7,7% of total g/day |
| Llavero-Valero, 2021 | Diabetes | Navarra (SUN project) | UPFs intake as g/day | 136-item FFQ | They tended to snack more, consume more soft drinks | 12 years | 20060 (175) | >323.3 g/d | <214.6 g/d |
| Nardocci,  2021 | Diabetes | Canada | UPFs intake as (% kcal/day) | 24-h dietary recalls | Not reported |  | 13608 | ≥ 58.7% kcal/day | ≤ 38.5% kcal/day |
| Nardocci,  2021 | Hypertension | Canada | UPFs intake as (% kcal/day) | 24-h dietary recalls | Not reported |  | 13556 | ≥ 58.7% kcal/day | ≤ 38.5% kcal/day |
| Nardocci,  2021 | Obesity | Canada | UPFs intake as (% kcal/day) | 24-h dietary recalls | Not reported |  | 13556 (3606) | ≥ 58.7% kcal/day | ≤ 38.5% kcal/day |
| Srour,  2020 | Diabetes | France (NutriNet-Santé study) |  | 3 non-consecutive validated web-based 24-hour dietary records at baseline and every 6 months (to vary the season of completion), randomly assigned over a 2-week period (2 weekdays and 1 weekend day) | Main UPF groups consumed were sugary products (28%) followed by ultra processed fruits and vegetables (18%), beverages (16%), starchy foods and breakfast cereals (11%), and processed meat and fish (11%) | 6 years | 104707 (821) |  |  |
| Shim,  2022 | Hypertension | Korean | UPFs intake as (%) | 24-h dietary recall | Not reported |  | 9188 |  |  |

| **Authors,**  **years** | **Outcome** | **Coutry/**  **Ethnicity** | **Type of foods** | **Dietary intake assessment: method** | **Main food contibutors** | **Follow-up (years)** | **Total population (number of cases)** | **Higher exposure** | **Lower exposure** |
| --- | --- | --- | --- | --- | --- | --- | --- | --- | --- |
| Scaranni,  2021 | Hypertension | Brasil | UPFs intake as (%) | 114-item FFQ | consumed no alcohol, had a higher intake of saturated fats | 3·9 years | 8754 (1312) | high (28·9–73·8 %) | low (0·1–20·5 %) |
| Ivancovsky-Wajcman,  2021 | Hypertension | Israel | (Kcal UPF/total kcal) | 117-item FFQ | They tended to consume more calories, with a higher proportion of calories from SFAs and carbohydrates, and a lower proportion from protein. Also, subjects with high UPF consumption drunk significantly less coffee and tended to eat less fibre | Cross-sectional | 789 (495) | > median 28% | < median 28% |
| Ivancovsky-Wajcman,  2021 | Low-HDL-c/ Hypertriglyceridemia | Israel | (Kcal UPF/total kcal) | 117-item FFQ | They tended to consume more calories, with a higher proportion of calories from SFAs and carbohydrates, and a lower proportion from protein. Also, subjects with high UPF consumption drunk significantly less coffee and tended to eat less fibre | Cross-sectional | 789 (495) | > median 28% | < median 28% |
| Rezende-Alves, 2020 | Hypertension | Brasil | UPFs intake as (%) | 144-item FFQ | Not reported | 2 years | 1221 (370) | 34.6–76.2% | 0.8–16.6% |
| Scaranni,  2023 | Hypertriglyceridemia | Brasil | UPFs intake as (weight %) | 114-item FFQ | Participants with higher consumption of UPF showed significantly higher consumption of saccharose, added sugar, total fats, saturated fat, unsaturated fat and trans-fats, besides lower consumption of fibre and n-3 fatty acids | 3·9 years | 5275 (857) | high 20.4–72.4 % | low 0.40–12.4 % |
| Scaranni,  2023 | Low-HDL-c | Brasil | UPFs intake as (weight %) | 114-item FFQ | Participants with higher consumption of UPF showed significantly higher consumption of saccharose, added sugar, total fats, saturated fat, unsaturated fat and trans-fats, besides lower consumption of fibre and n-3 fatty acids | 3·9 years | 5275 (842) | high 20.4–72.4 % | low 0.40–12.4 % |
| Donat-Vargas, 2021 | Low-HDL-c | Spain | UPFs intake as (%) | Validated computer-based dietary history | The UPF groups that contributed most to the quantity of UPFs consumed were the  following: cookies and pastries (31.2%), processed meat and meat products (15.7%), breakfast cereals and breads (11.1%), and sweets (10.9%), among others | 5–7 years | 1082 (878) | 31.4 ± 8 % of energy | 7.60 ± 3.6 % of energy |

| **Authors,**  **years** | **Outcome** | **Coutry/**  **Ethnicity** | **Type of foods** | **Dietary intake assessment: method** | **Main food contibutors** | **Follow-up (years)** | **Total population (number of cases)** | **Higher exposure** | **Lower exposure** |
| --- | --- | --- | --- | --- | --- | --- | --- | --- | --- |
| Donat-Vargas, 2021 | Hypertriglyceridemia | Spain | UPFs intake as (%) | Validated computer-based dietary history | The UPF groups that contributed most to the quantity of UPFs consumed were the  following: cookies and pastries (31.2%), processed meat and meat products (15.7%), breakfast cereals and breads (11.1%), and sweets (10.9%), among others | 5–7 years | 1082 (895) | 31.4 ± 8 % of energy | 7.60 ± 3.6 % of energy |
| Donat-Vargas, 2021 | High-LDL-c | Spain | UPFs intake as (%) | Validated computer-based dietary history | The UPF groups that contributed most to the quantity of UPFs consumed were the  following: cookies and pastries (31.2%), processed meat and meat products (15.7%), breakfast cereals and breads (11.1%), and sweets (10.9%), among others | 5–7 years | 1082 (472) | 31.4 ± 8 % of energy | 7.60 ± 3.6 % of energy |
| Haghighatdoost, 2022 | Obesity | Iran | UPFs intake as (% kcal/day) | 136-item FFQ | Fast food dishes (Hamburgers, hot dogs, fries, pizzas, sandwiches, and other products bought in fast food outlets); others (Canned soups, baby products, canned mixed dishes, cheese products, frozen and prepared french-fries and onion rings, fish or seafood imitations, meal replacements, sweeteners, protein shake powder, egg substitutes, coffee whitener, veggie slice, vanilla extract, malt extract,  whey protein, added calcium, and soy protein); Sweetened milk-based products (Ice cream, chocolate milk, flavored yogurt, milkshakes, and malted milk); Soft drinks, sweetened fruit juices, and drinks | Cross-sectional | 1459 (329) | 36.2% of energy | 6.9% of energy |
| Cordova,  2021 | Obesity | EPIC study 10 European countries: Denmark, France, Germany, Greece, Italy, the Netherlands, Norway, Spain, Sweden, and the United Kingdom | UPFs intake as g/day | country-specific validated dietary  questionnaires | Participants in the highest quintile consumed more soft drinks and less alcohol compared to those in the lowest quintile | median follow-up time of 5 years | 348748 (103259) | 686 ± 303 | 176 ± 102 |

| **Authors,**  **years** | **Outcome** | **Coutry/**  **Ethnicity** | **Type of foods** | **Dietary intake assessment: method** | **Main food contibutors** | **Follow-up (years)** | **Total population (number of cases)** | **Higher exposure** | **Lower exposure** |
| --- | --- | --- | --- | --- | --- | --- | --- | --- | --- |
| Rauber,  2021 | Obesity | UK | UPF intake as % of total energy | 24-h dietary recall | The main food groups contributing to UPFs intake were snacks and  desserts (33%, for example, pastries, buns, cakes, biscuits, confectionary, packaged salty snacks and industrial desserts) followed by ultra-processed bread (21%, for example, bagel, burger bun, bread roll and bap), frozen and shelfstable ready-to-eat/heat meals (16%, for example, industrial chips/French fries, sausage, nuggets, fish fingers and other reconstituted meat products, industrial pizza and packaged pre-prepared meals), beverages (15%, for example, milkbased drinks, soft and fruit drinks, fruit juices, alcoholic drinks and coffee drinks), spreads, sauces and other UPFs (9%, margarine and other spreads, sauces, dressing and gravies, chocolate/nut spread, spreadable cheese, sweeteners and meat alternative), and breakfast cereals (6%, for example, sweetened cornflake and sweetened oat crunch type cereal) | 5.6 years | 17271 (947) | 71.5% | 25.5% |
| Li,  2021 | Obesity | China | UPF intake as (g/day) | 24-h dietary recall on each of three consecutive days | Higher consumers (50 g/d) had higher intake of energy, fat and protein but lower carbohydrates | 12 years | 12451 (2211) | >50 g/d | non-consumers |
| Sung, 2021 | Obesity | Korean adults | UPF intake as % of total energy | 24-h dietary recall data | Among UPFs, beverages such as carbonated drinks, fruit drinks, and  coffee (4.3%); industrialized bread, cakes, and bakery products (3.6%); sauce, dressing, and condiments (3.5%); and instant noodles (3.5%) were the highest contributors to total energy consumption |  | 7364 | 39 | 12.5 |
| Pestoni,  2021 | Obesity | 10 study centers across Switzerland | UPF intake as (% weight) | two 24-hour dietary recalls | Not reported | Cross-sectional | 2057 (265) | 40% | 10% |
| Machado, 2020 | Obesity | Australia | UPF intake as % of total energy | two non-consecutive 24-h dietary recalls | Not reported |  | 7411 (1964) | 74.2% (range  62.1–100%) in the highest quintile | 12.7% (range 0–21.7%) in the lowest quintile |

| **Authors, years** | **Outcome** | **Coutry/**  **Ethnicity** | **Type of foods** | **Dietary intake assessment: method** | **Main food contibutors** | **Follow-up (years)** | **Total population (number of cases)** | **Higher exposure** | **Lower exposure** |
| --- | --- | --- | --- | --- | --- | --- | --- | --- | --- |
| Beslay, 2020 | Obesity | France (NutriNet-Santé study) | UPF intake as (% weight of g/day) | 3 non-consecutive web-based 24-h records, randomly assigned over a 2-week period (2 during weekdays and 1 during the weekend) | They had also higher sodium, sugar, SFA, and energy intake and lower intakes of dietary fibre and alcohol | 5.0  years | 71871 (3066) | 32.4 (9.6) | 7.5 (2.1) |
| Rauber, 2020 | Obesity | UK | UPF intake as (% of total energy) | 4 day food diary | Ultra-processed breads; Packaged pre-prepared meals (Including frozen and shelf-stable dishes and canned soups); Breakfast cereals; Sausage and other reconstituted meat products; Confectionary; biscuits; Pastries, buns, and cakes; Soft drinks, fruit drinks and fruit juices |  | 6143 | 74% (4th quartile) | 35% (1st quartile) |
| Canhada, 2019 | Obesity | Brasil | UPF intake as (% of total energy | FFQ with 114 food items | Bread, sweets/candies, sweetened sodas/juices and salty pastries/chips accounted formore than 50% of the total energy consumption from ultra-processed foods; other frequent items (>5%) included cakes, processed meat, pasta/pizzas, cookies/crackers and mayonnaise/margarine/cream cheese |  | 11827 (972) | 30.84–73.84 % | 0–17.79 % |
| Juul, 2018 | Obesity | US | UPF intake as (% of total energy | Two 24-h dietary recall interviews | participants consuming the most UPFs (quintile 5) had higher average energy intake, greater relative energy contributions from carbohydrates, sugar, SFA and PUFA and lower relative intake of protein and fibre |  | 15977 (5768) | ≥74.2% | ≤36.5% |
| Marcelina Silva, 2018 | Obesity | Brasil | UPF intake as 7% of total energy intake | semi-quantitative FFQ with 114 food items | Not reported |  | 8977 (1768) | >29% | <16% |

**Supplementary Table 2.** Newcastle - Ottawa quality assessment scale cohort studies (Diabetes).

|  | Representativeness of the exposed cohort | Selection of the non exposed cohort | Ascertainment of exposure | Demonstration that outcome of interest was not present at start of study | Comparability of cohorts | Assessment of outcome | Length of follow-up | Lost at follow up | Total |
| --- | --- | --- | --- | --- | --- | --- | --- | --- | --- |
| Duan 2022 | **🟑** | **🟑** | **🟑** | **🟑** | **🟑🟑** | **🟑** | **🟑** | **🟑** | 9/9 |
| Sen 2022 | **🟑** | **🟑** | **🟑** | **🟑** | **🟑** |  | **🟑** | **🟑** | 7/9 |
| Li 2022 | **🟑** | **🟑** | **🟑** | **🟑** | **🟑** | **🟑** | **🟑** | **🟑** | 8/9 |
| Srour 2019 | **🟑** | **🟑** | **🟑** | **🟑** | **🟑** | **🟑** | **🟑** | **🟑** | 8/9 |
| Levy 2021 | **🟑** | **🟑** | **🟑** | **🟑** | **🟑** |  | **🟑** | **🟑** | 7/9 |
| Llavero-Valero 2021 | **🟑** | **🟑** | **🟑** | **🟑** | **🟑🟑** | **🟑** | **🟑** | **🟑** | 9/9 |
| Nardocci 2021 | **🟑** | **🟑** | **🟑** | **🟑** | **🟑🟑** | **🟑** | **🟑** | **🟑** | 9/9 |

**Supplementary Table 2.** Cont. (hypertension)

|  | Representativeness of the exposed cohort | Selection of the non exposed cohort | Ascertainment of exposure | Demonstration that outcome of interest was not present at start of study | Comparability of cohorts | Assessment of outcome | Length of follow-up | Lost at follow up | Total |
| --- | --- | --- | --- | --- | --- | --- | --- | --- | --- |
| Shim 2022 | **🟑** | **🟑** | **🟑** | **🟑** | **🟑** | **🟑** | **🟑** | **🟑** | 8/9 |
| Ivancovsky-Wajcman 2021 | **🟑** | **🟑** | **🟑** | **🟑** | **🟑🟑** | **🟑** |  | **🟑** | 8/9 |
| Nardocci 2021 | **🟑** | **🟑** | **🟑** | **🟑** | **🟑🟑** | **🟑** |  |  | 7/9 |
| Scaranni 2021 | **🟑** | **🟑** | **🟑** | **🟑** | **🟑** | **🟑** | **🟑** | **🟑** | 8/9 |
| Rezende-Alves 2020 | **🟑** | **🟑** | **🟑** | **🟑** | **🟑** | **🟑** | **🟑** | **🟑** | 8/9 |

**Supplementary Table 2.** Cont. (Dyslipidemia)

|  | Representativeness of the exposed cohort | Selection of the non exposed cohort | Ascertainment of exposure | Demonstration that outcome of interest was not present at start of study | Comparability of cohorts | Assessment of outcome | Length of follow-up | Lost at follow up | Total |
| --- | --- | --- | --- | --- | --- | --- | --- | --- | --- |
| Scaranni 2022 | **🟑** | **🟑** | **🟑** | **🟑** | **🟑** | **🟑** | **🟑** | **🟑** | 8/9 |
| Donat-Vargas 2021 | **🟑** | **🟑** | **🟑** | **🟑** | **🟑** | **🟑** | **🟑** | **🟑** | 8/9 |
| Ivancovsky-Wajcman 2021 | **🟑** | **🟑** | **🟑** | **🟑** | **🟑🟑** | **🟑** |  | **🟑** | 8/9 |

**Supplementary Table 2.** Cont. (Low-HDL)

|  | Representativeness of the exposed cohort | Selection of the non exposed cohort | Ascertainment of exposure | Demonstration that outcome of interest was not present at start of study | Comparability of cohorts | Assessment of outcome | Length of follow-up | Lost at follow up | Total |
| --- | --- | --- | --- | --- | --- | --- | --- | --- | --- |
| Donat-Vargas 2021 | **🟑** | **🟑** | **🟑** | **🟑** | **🟑** | **🟑** | **🟑** |  | 7/9 |
| Ivancovsky-Wajcman 2021 | **🟑** | **🟑** | **🟑** | **🟑** | **🟑🟑** | **🟑** | **🟑** | **🟑** | 9/9 |
| Scaranni 2022 | **🟑** | **🟑** | **🟑** | **🟑** | **🟑🟑** | **🟑** | **🟑** | **🟑** | 9/9 |

**Supplementary Table 2.** Cont. ( Obesity)

|  | Representativeness of the exposed cohort | Selection of the non exposed cohort | Ascertainment of exposure | Demonstration that outcome of interest was not present at start of study | Comparability of cohorts | Assessment of outcome | Length of follow-up | Lost at follow up | Total |
| --- | --- | --- | --- | --- | --- | --- | --- | --- | --- |
| Beslay 2020 | **🟑** | **🟑** | **🟑** | **🟑** | **🟑** | **🟑** | **🟑** | **🟑** | 8/9 |
| Canhada 2019 | **🟑** | **🟑** | **🟑** | **🟑** | **🟑** | **🟑** | **🟑** |  | 7/9 |
| Cordova 2021 | **🟑** | **🟑** | **🟑** | **🟑** | **🟑** | **🟑** | **🟑** | **🟑** | 8/9 |
| Haghighatdoost 2022 | **🟑** | **🟑** | **🟑** | **🟑** | **🟑** | **🟑** | **🟑** | **🟑** | 8/9 |
| Juul 2018 | **🟑** | **🟑** | **🟑** | **🟑** | **🟑🟑** | **🟑** | **🟑** | **🟑** | 9/9 |
| Li 2021 | **🟑** | **🟑** | **🟑** | **🟑** | **🟑** | **🟑** | **🟑** | **🟑** | 8/9 |
| Machado 2020 | **🟑** | **🟑** | **🟑** | **🟑** | **🟑** | **🟑** | **🟑** |  | 7/9 |
| Nardocci 2021 | **🟑** | **🟑** | **🟑** | **🟑** | **🟑** | **🟑** | **🟑** | **🟑** | 8/9 |
| Pestoni 2021 | **🟑** | **🟑** | **🟑** | **🟑** | **🟑** | **🟑** | **🟑** | **🟑** | 8/9 |
| Rauber 2020 | **🟑** | **🟑** | **🟑** | **🟑** | **🟑** | **🟑** | **🟑** | **🟑** | 8/9 |
| Rauber 2021 | **🟑** | **🟑** | **🟑** | **🟑** | **🟑** | **🟑** | **🟑** |  | 7/9 |
| Silva 2018 | **🟑** | **🟑** | **🟑** | **🟑** | **🟑** | **🟑** | **🟑** |  | 7/9 |
| Sung 2021 | **🟑** | **🟑** | **🟑** | **🟑** | **🟑** | **🟑** | **🟑** | **🟑** | 8/9 |

**Supplementary Table 3.** NutriGrade scoring system

| **Study ID** | **Outcome** | **Risk of bias, study quality, and study limitations** | **Precision** | **Heterogeneity** | **Directness** | **Publication bias** | **Funding bias** | **Effect size** | **Dose-response** | **Total** | **Media** | **Quality of evidence** |
| --- | --- | --- | --- | --- | --- | --- | --- | --- | --- | --- | --- | --- |
| Duan, 2022 | Diabetes | 1,5 | 1 | 0 | 1 | 0,5 | 1 | 1 | 1 | 7 | 6,21 | Moderate |
| Li, 2022 | Diabetes | 2 | 0 | 0 | 1 | 0,5 | 1 | 1 | 0 | 5,5 |  |  |
| Sen, 2022 | Diabetes | 2 | 0 | 0 | 1 | 0,5 | 1 | 1 | 0 | 5,5 |  |  |
| Levy, 2021 | Diabetes | 2 | 0 | 0 | 1 | 0,5 | 1 | 1 | 1 | 6,5 |  |  |
| Llavero-Valero, 2021 | Diabetes | 2 | 0 | 0 | 1 | 0,5 | 1 | 1 | 0 | 5,5 |  |  |
| Nardocci, 2021 | Diabetes | 1,5 | 0 | 0 | 1 | 0,5 | 1 | 1 | 1 | 6 |  |  |
| Srour, 2020 | Diabetes | 2 | 1 | 0 | 1 | 0,5 | 1 | 1 | 1 | 7,5 |  |  |
| Shim, 2022 | Hypertension | 2 | 0 | 0 | 1 | 0,5 | 1 | 1 | 0 | 5,5 | 5,50 | Low |
| Nardocci, 2021 | Hypertension | 1,5 | 0 | 0 | 1 | 0,5 | 1 | 1 | 1 | 6 |  |  |
| Scaranni, 2021 | Hypertension | 1,5 | 1 | 0 | 1 | 0,5 | 1 | 1 | 0 | 6 |  |  |
| Ivancovsky-Wajcman, 2021 | Hypertension | 1,5 | 0 | 0 | 1 | 0,5 | 1 | 1 | 0 | 5 |  |  |
| Rezende-Alves, 2020 | Hypertension | 1,5 | 0 | 0 | 1 | 0,5 | 1 | 1 | 0 | 5 |  |  |
| Scaranni, 2022 | Hypertriglyceridemia | 1,5 | 1 | 0 | 1 | 0 | 1 | 1 | 0 | 5,5 | 5,67 | Low |
| Scaranni, 2022 | Low-HDL-c | 1,5 | 1 | 0 | 1 | 0 | 1 | 1 | 0 | 5,5 |  |  |
| Ivancovsky-Wajcman, 2021 | Low-HDL-c | 1,5 | 0 | 0 | 1 | 0 | 1 | 1 | 0 | 4,5 |  |  |
| Ivancovsky-Wajcman, 2021 | Hypertriglyceridemia | 1,5 | 0 | 0 | 1 | 0 | 1 | 1 | 0 | 4,5 |  |  |
| Donat-Vargas, 2021 | Low-HDL-c | 2 | 1 | 0 | 1 | 0 | 1 | 1 | 1 | 7 |  |  |
| Donat-Vargas, 2021 | Hypertriglyceridemia | 2 | 1 | 0 | 1 | 0 | 1 | 1 | 1 | 7 |  |  |
| Haghighatdoost, 2022 | Obesity | 1,5 | 0 | 0 | 1 | 0,5 | 1 | 1 | 0 | 5 | 6,00 | Low |
| Nardocci, 2021 | Obesity | 1,5 | 1 | 0 | 1 | 0,5 | 1 | 1 | 0 | 6 |  |  |
| Cordova, 2021 | Obesity | 2 | 1 | 0 | 1 | 0,5 | 1 | 1 | 0 | 6,5 |  |  |
| Rauber, 2021 | Obesity | 2 | 1 | 0 | 1 | 0,5 | 1 | 1 | 1 | 7,5 |  |  |
| Li, 2021 | Obesity | 2 | 1 | 0 | 1 | 0,5 | 1 | 1 | 0 | 6,5 |  |  |
| Sung, 2021 | Obesity | 1,5 | 0 | 0 | 1 | 0,5 | 1 | 1 | 0 | 5 |  |  |
| Pestoni, 2021 | Obesity | 1,5 | 0 | 0 | 1 | 0,5 | 1 | 1 | 0 | 5 |  |  |
| Machado, 2020 | Obesity | 1,5 | 1 | 0 | 1 | 0,5 | 1 | 1 | 0 | 6 |  |  |
| Beslay, 2020 | Obesity | 2 | 1 | 0 | 1 | 0,5 | 1 | 1 | 0 | 6,5 |  |  |
| Rauber, 2020 | Obesity | 1,5 | 0 | 0 | 1 | 0,5 | 1 | 1 | 1 | 6 |  |  |
| Canhada, 2019 | Obesity | 1,5 | 1 | 0 | 1 | 0,5 | 1 | 1 | 0 | 6 |  |  |
| Juul, 2018 | Obesity | 1,5 | 1 | 0 | 1 | 0,5 | 1 | 1 | 0 | 6 |  |  |
| Silva, 2018 | Obesity | 1,5 | 1 | 0 | 1 | 0,5 | 1 | 1 | 0 | 6 |  |  |

**Supplementary Figure 1.** Funnel plots of the log RR versus the standard error for studies evaluating associations between UPFs and the risk of metabolic disorders A) Diabetes B) Hypertension C) Hypertriglyceridemia D) Low HDL-cholesterol E) Obesity

A

C

B


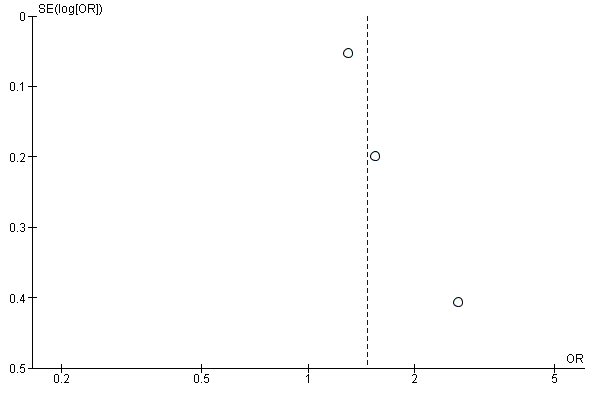

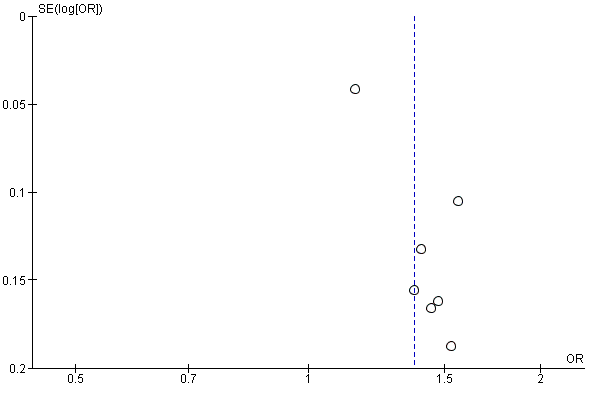

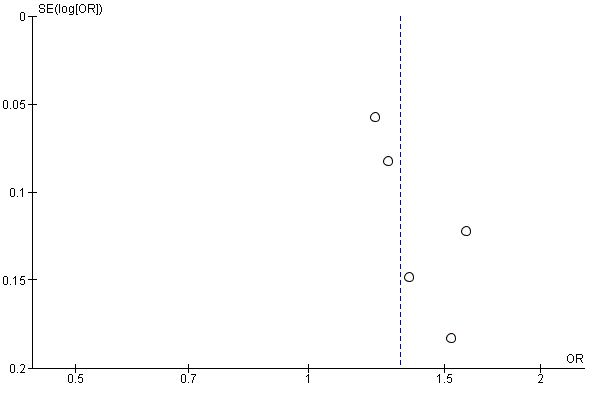


E

D

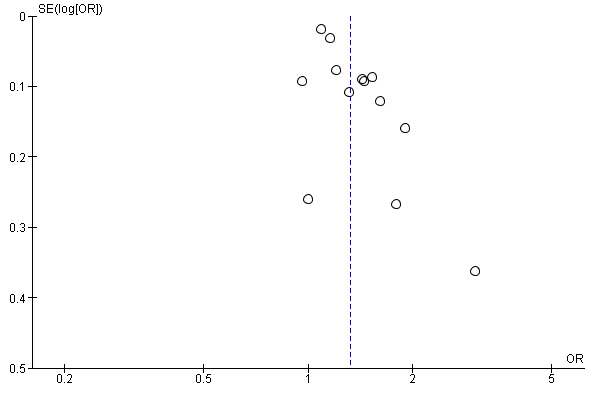


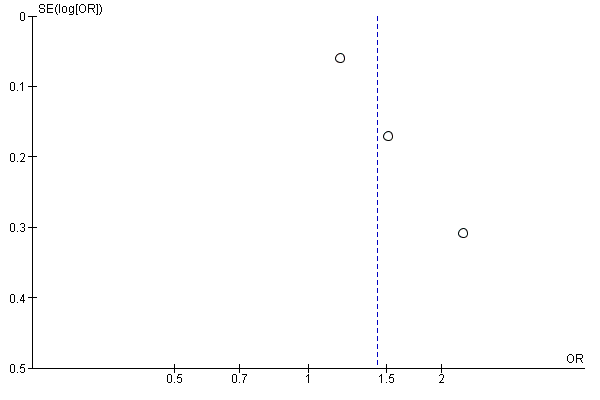

Supplement: Multimedia component1 [file mmc1.docx]
